# Body Massage Performance Investigation by Brain Activity Analysis

**DOI:** 10.1155/2012/252163

**Published:** 2012-03-19

**Authors:** Kang-Ming Chang, Shu-Yi Luo, Sih-Huei Chen, Tuan-Ping Wang, Congo Tak-Shing Ching

**Affiliations:** ^1^Department of Photonics and Communication Engineering, Asia University, Wufeng, Taichung 41354, Taiwan; ^2^Graduate Institute of Clinical Medical Science, China Medical University, Taichung 40402, Taiwan; ^3^Department of Creative Product Design, Asia University, Wufeng, Taichung 41354, Taiwan; ^4^Department of Computer Science and Information Engineering, Asia University, Wufeng, Taichung 41354, Taiwan; ^5^Department of Industrial Engineering and Engineering Management, National Tsing Hua University, Hsinchu 30013, Taiwan; ^6^Department of Electrical Engineering, National Chi Nan University, Puli, Nantou 54561, Taiwan

## Abstract

Massage has been widely applied to improve health and reduce stress. However, the performance difference between hands-on treatment and treatment by mechanical devices has been little mentioned. Therefore, the main aim of this paper is to investigate a subject's EEG performance under massage treatment applied by hand and treatment applied by mechanical devices. Massage was applied to four acupoints for three minutes each. The massage acupoint sequence was from left Jian-wai-yu, right Jian-wai-yu, left Zuo-fei-yu, and finally right Zuo-fei-yu. An EEG system of 32 channels was used. Twenty-four volunteers, mainly college students, were enrolled. EEG rhythm powers of each massage sessions were derived. Two-way ANOVA revealed that there were also significant interactions between the massage stage and the massage type on delta (*P* < 0.01), theta (*P* < 0.05), and beta rhythms (*P* < 0.01), and there were significant differences at different stages for the mechanical massage group (*F* = 5.557, *P* < 0.01). The mechanical massage group had more significant differences than the hands-on group for stage coherence of around coherence on alpha rhythm. Further rhythm power scalp topography between two massage methods is also investigated.

## 1. Introduction

Massage is considered to be a complementary and alternative medicinal (CAM) therapy [1, 2]. Massage has also had a long history of being widely used to improve health in countries such as China, India, and Egypt [3, 4]. Massage can eliminate muscle soreness and prevent exercise injury. Massage can also make skin shiny, flexible, and can even cure some diseases without medication [5, 6]. Hippocrates, the Father of Medicine, was the first to mention the importance of massage in the treatment in western medicine. Hippocrates considered “massage” or the friction (rubbing) as “a healthy gear (wheel of health essentials)” [7]. There are five techniques mainly used during massage: stroking, compression, friction, vibration, and percussion [8]. The American Massage Therapy Association (AMTA) defines massage as “manual soft tissue manipulation, including holding, causing movement, and/or applying pressure to the body” [2]. There are many articles that investigate the effects of massage. The stress reduction performance of massage based on physiological measures has been fully reviewed by Moraska et al. [9]. The effectiveness of massage on a variety of chronic, nonmalignant pain complaints has also been reviewed by Jennie [10]. Dibble et al. have investigated massage on finger acupoints among women undergoing chemotherapy for breast cancer and noted a reduced nausea intensity [11]. Massage has also been widely applied to infants to improve their health [12] and also for climacteric women [13]. Abundant research that focuses on pressure, massage method, acupoints, and also other methods, such as aromatherapy, indicates an increase in the immune system [14]. Hatayama studied face massage by HRV for adult women and noticed a significant increase of LF/HF after massage, that means an increase in the sympathetic nervous system [15]. The effects of facial massage and foot massage were also compared. Results showed that face massage was marginally better at producing subjective sleepiness than foot massage [16].

**Figure 1:**
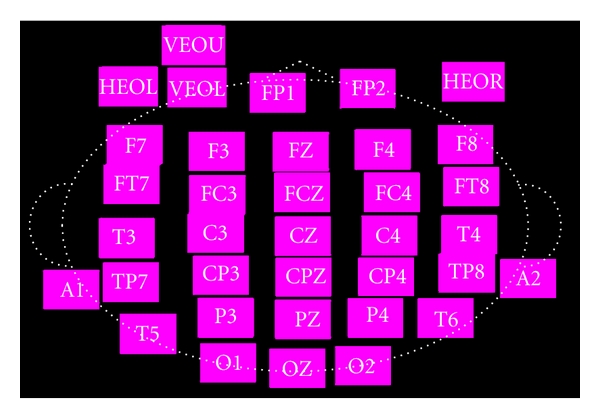
Standard 10–20 system for EEG electrode label.

It is very important to understand the subject's feelings during massage therapy. A comfortable feeling is expected after massage therapy. Therefore, Electroencephalography (EEG) is used as a physiological tool to reflect the brain state during massage. EEG is derived from electrical charge movement between nerve cell membranes within the cerebral cortex, and EEG signals are often characterized by frequency range. Traditionally, four major EEG rhythms are used. These are *δ* waves (<4 Hz), *θ* waves (4–8 HZ), *α* waves (8–13 Hz), and *β* waves (13–25 Hz). EEG also indicates brain state within different brain regions. According to the 10–20 system, each EEG electrode has been named with a combination of a letter and a number. The left brain region being odd numbered, and right brain region being even numbered. The “F” character represents the frontal lobe, “C” is for the central region, “P” is the parietal lobe, “T” is the temporal lobe, and “O” is the occipital lobe. The distribution of EEG electrodes is illustrated in Figure 1 [17]. Jones and Field [18], Diego et al. [19], and Field et al. [20, 21] have investigated massage by EEG for many years. They have discussed the impact of massage on pressure relief in operations research and awareness with EEG. Fifty subjects were divided into two groups, one group was treated with massage chairs, and the other group was treated by professional massage technicians. Results showed that massage groups treated by professional technicians had significantly decreased *α* and *β* waves on the frontal lobe. This indicates that professional technicians' massage can reduce tension and induce relaxation.

Asymmetry EEG powers may be another important factor reflecting happiness during massage. According to Davidson's hypothesis of affective frontal asymmetry, prefrontal brain systems are regions of emotional excitation. The left prefrontal brain region is composed mainly of positive emotions, while the right anterior frontal brain region is mainly for negative emotions, such as withdrawal, sadness and fear [22, 23]. Different brain wave frequency regions correspond to different emotional states. Therefore, comparing the difference between the average powers of specific EEG rhythms can help to infer the psychological state of individuals [24]. Jones also applied the EEG asymmetry concept to infant massage [25]. They found that there was a significantly decreased EEG power in the right frontal lobe region, which means reduced anxiety and depression, according to Davidson's hypothesis. Based on the above findings, the human brain prefrontal cortex activity is highly related to emotional states and represented by the difference of alpha EEG power. This research also attempts to use frontal EEG alpha asymmetry as a physiological index to investigate the happiness state during massage.

Coherence value is another interesting index in EEG analysis. Coherence is a linear spatial relationship between two EEG channels, and it is widely used in the analysis of brain waves. The signal is very common [26, 27]. This study also adopts this method to assess spatial relationship during massages.

In Chinese massage, single or multiple parts of the body were processed by therapists. These specific parts of the body are called acupoints. Different acupoints have different effects on the subject's health [28, 29]. In addition to traditional hands-on treatment, mechanical massage devices have been used by therapists during massage therapy [10]. The massage performance difference between hands-on treatment and mechanical devices has been little mentioned, and the main aim of this paper is to investigate the subject's EEG performance under massage by hands-on treatment and massage by mechanical devices.

## 2. Method

### 2.1. Subjects and Experimental Design

**Figure 2:**
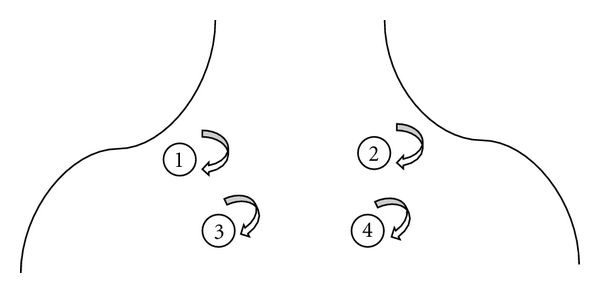
Acupoints used in this study. The massage sequences are from (1) left Jian-wai-yu, (2) right Jian-wai-yu, (3) left Zuo-fei-yu, and (4) right Zuo-fei-yu. Acupoints were identified by a professional massage therapist for both experimental groups.

**Figure 3:**
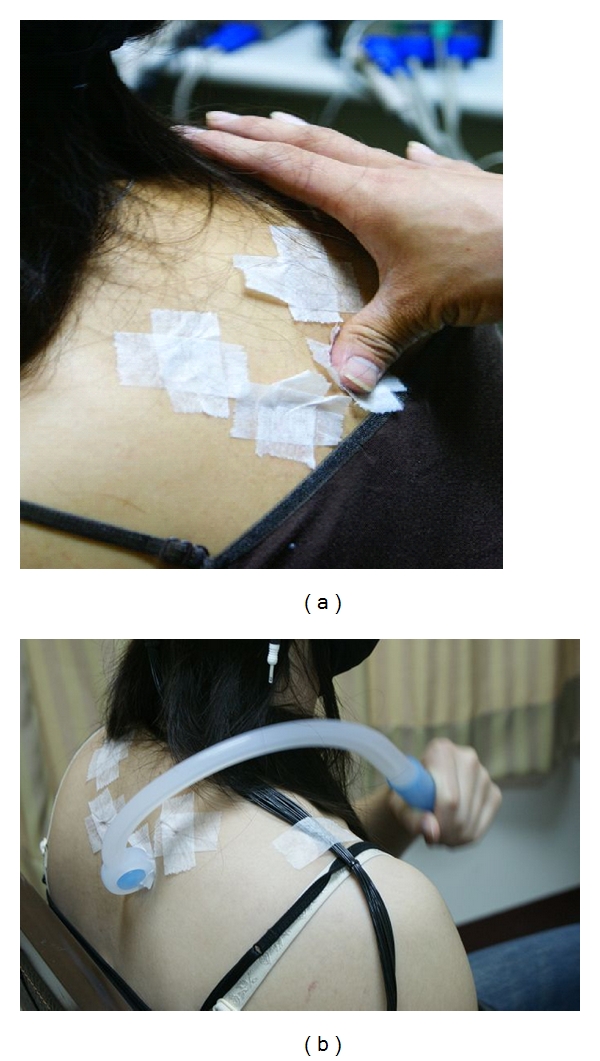
Illustration of massage method by (a) hand stimulus and (b) mechanical stimulus.

Twenty-four volunteers, mainly college students, were enrolled: thirteen male and eleven female, age range eighteen to twenty-four (mean age 23.3 and standard deviation 1.2). All subjects were required to take regular sleep and avoid smoking and drinking coffee. They were also no known heart- or muscle-related diseases. The EEG data were recorded in Asia University, Taiwan, by a 32-channel Synamps system produced by NeuroScan Inc. Subjects were sitting in a chair in a comfortable posture, and the massage was conducted by the same professional massage therapist. There were three sessions comprising three-minute presessions for baseline EEG recording (denoted as PRE); during the massage four acupoints were conducted with each three-minute massage in a clockwise direction. The massage acupoint sequence was from left Jian-wai-yu, right Jian-wai-yu, left Zuo-fei-yu, and finally right Zuo-fei-yu, as illustrated in Figure 2. These four sessions were denoted as D1, D2, D3, and D4. After the massage, EEG at three minutes and at six minutes was used as comparison (these postmassage sessions were denoted as P3 and P6). Subjects were divided into two groups by their own consent, one was the hand massage group (seven males and five females), and the other was a mechanical massage group (six males and six females). The hand massage was conducted mainly by means of the therapist's thumb, using moderate pressure, as demonstrated in Figure 3(a). Mechanical massage was conducted by the subjects themselves with the aid of the designed massage equipment, as shown in Figure 3(b). Subjects practiced using the massage equipment several times under the direction of the therapist before the EEG recordings were made. Massage pressure was moderate and similar to the pressure produced by the therapist. Hands-on massage of all the subjects was conducted by the same therapist. The same therapist also marked acupoints for hands-on and mechanical massage sessions. Each massage experiment interval was more than three hours, in order to be fully rested for the therapist. IRB was approved by Asia University Medical Research Ethics Committee.

### 2.2. EEG Signal Acquirement

EEG data was recorded via unipolar connection, with Fpz as ground and A1/A2 as reference points. A total thirty channels of EEG were recorded, inclusive of FP1, FP2, F7, F8, F3, F4, FC3, FC4, FT7, FT8, FZ, FCZ, T3, T4, T5, T6, TP7, TP8, C3, C4, CP3, CP4, CZ, CPZ, P3, P4, PZ, O1, O2, and Oz. EEG channel impedance was adjusted until it measured below 5 kΩ. A band-pass filter of 0.05–50 Hz was applied during recording. Sampling frequency was 1000 Hz. Afterwards offline analysis was first processed by an IIR band-pass filter between 1–30 Hz with zero phase shift filtering and 12 dB/Oct. EEG epoch was with intervals of 1024 sampling points. After baseline correction and artifact rejection, frequency domain features were extracted by FFT with a 1024-point Hanning window. Four EEG rhythm powers were calculated by different frequency range: *δ*(0.5~4 Hz), *θ*(4~8 Hz), *α*(8~13 Hz), *β*(13~30 Hz).

### 2.3. EEG Signal Analysis

EEG rhythm power and coherence were derived from the SCAN system. The mathematical expression of coherence is shown in(1)

(1) $${\text{coherence}}_{x,y}\left(f\right)=\frac{P_{x,y}\left(f\right)}{\sqrt{\left|P_{x,x}\left(f\right)\right|}\sqrt{\left|P_{y,y}\left(f\right)\right|}},$$

where *P*_*xx*_(*f*), *P*_*yy*_(*f*), and *P*_*x*,*y*_(*f*), are power spectrum density (PSD), *f* is frequency range in terms of Hz, and PSD is defined as in(2)

(2) $$P_{x,y}\left(f\right)=\frac{\sum_{m=-\infty }^{\infty }R_{x,y}\left(m\right)e^{-(j2\pi fm/f_{s})}}{2\pi },$$

where *R*_*x*,*ym*_(*f*) is the cross-correlation function of EEG segment and *f*_*s*_ is sampling frequency. There are 30*30 coherence pairs for 30 EEG channels. In order to simplify analysis procedure, two groups of coherence pairs were used for analysis. The first group is to compare the front region with the hind brain region in the central line; six pairs were used: Fz-Cz, Fz-Pz, Fz-Oz, Cz-Pz, Cz-Oz, and Pz-Oz, known as around coherence. The second group is to compare the left brain region to the right brain region symmetrically. Five pairs were used, which were FP1-FP2, F3-F4, C3-C4, P3-P4, and O1-O2, known as symmetry coherence.

The power and coherence data were processed with Synamps software SCAN and exported to EXCEL for further asymmetry index calculation and statistical analysis. Taking natural logarithm to *α* power in the F3 and F4 channels, the difference between F4 and F3 powers is the alpha-asymmetry index. A positive index represents a positive emotion, while a negative index shows negative emotions [19].

### 2.4. Statistics

The SPSS 12.0 software package was used to conduct data analysis. Significance test for the alpha value was set at 0.05. The statistical methods used in this study are summarized as follows. 

#### 2.4.1. Descriptive Statistics

Personal information on subjects, and EEG power and coherence of different stage/frequency region are represented as mean ± standard deviation.

#### 2.4.2. Paired *t*-Test

Intragroup differences between different massage stages of the four EEG rhythm powers and coherence were examined by paired *t*-test.

#### 2.4.3. Two-Way ANOVA

To examine the interaction effect between the two massage methods and the seven massage stages by two-way ANOVA.

## 3. Results

**Figure 4:**
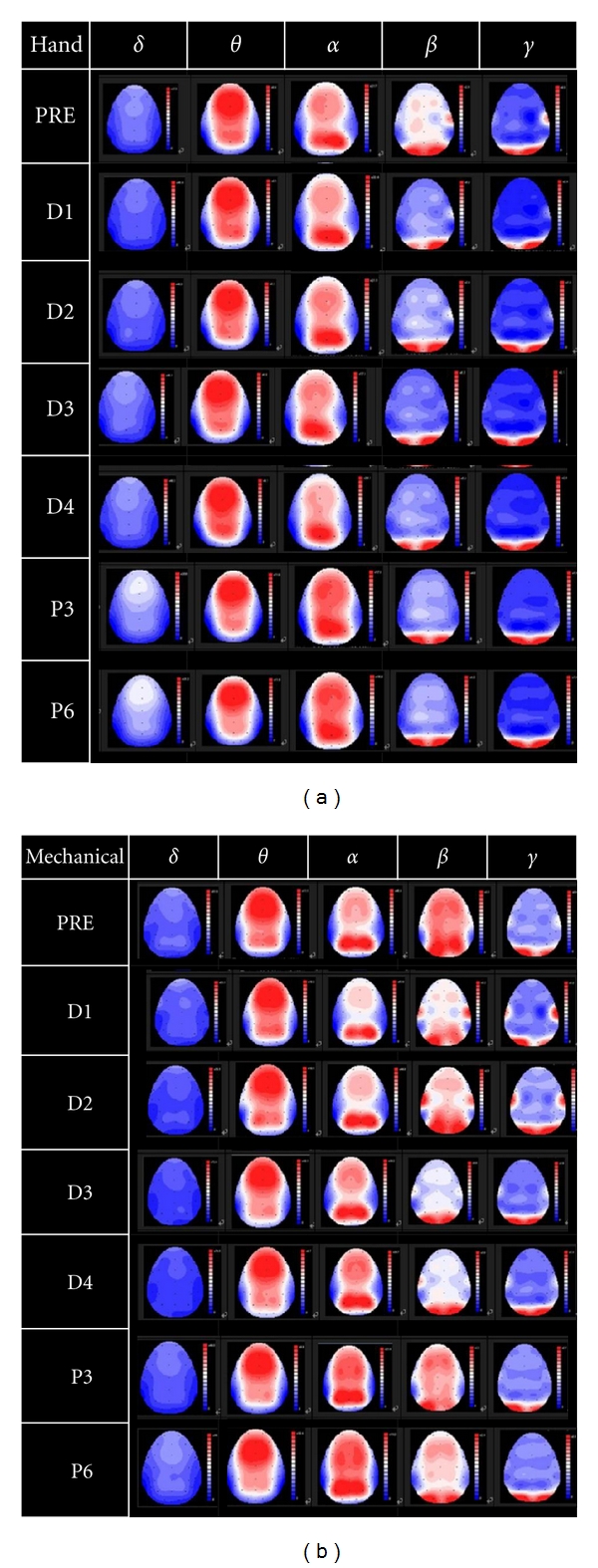
Average EEG power scalp topography: (a) hands-on group and (b) mechanical-based group.

The average of EEG power topography is shown in Figure 4 for the two groups. Similar to previous studies in moderate massage [19, 20, 30], there were increases in delta rhythm and decreases in alpha rhythm [31]. Detail sub-band EEG power distributions are listed in Table 1. Two-way ANOVA revealed that there was no significant difference between the two massage types; there were statistically significant differences for the seven massage stages on the alpha and beta bands. There were also significant interactions between the massage stage and the massage type on delta, theta, and beta rhythms. Detail EEG power variation of sub-band rhythm is as follows. For delta rhythm, there was an increase in power during massage for the hands-on group; while there was a decrease for the mechanical massage group. During phase P3, there was a decrease for the mechanical massage group and a gradual reduction for the hand massage group. For theta rhythm, there was an increase for the hand massage group, while there was gentle decrease for the mechanical massage group. After the massage, there were rising trends for both groups. For alpha rhythm, there was a gradual decrease in power during the massage session, especially for the mechanical massage group. For beta rhythm, there was an upward trend from PRE session to massage session, especially for the mechanical massage group. During P3 and P6 sessions, there was apparently a decrease in power for the mechanical massage group. Furthermore, main factor analysis showed that there are significant differences at different stages for the mechanical massage group (*F* = 5.557, *P* < 0.01).

Two-way ANOVA results for sub-band power of each EEG channels are listed in Table 2. For the delta rhythm, there were statistically significant differences on seventeen channels among thirty channels, with the interaction between massage stage and massage type; while there were no significant differences on stage and on massage type. There were six channels with significant stage-type interaction for theta rhythm, but there was no significant difference on stage or type. Theta rhythm seems not to be an effective rhythm during massage. Instead, there were seventeen channels having stage significance for alpha rhythm. But there were only four channels with type and stage-type interaction. There were also nineteen channels with significant stage and sixteen channels with stage-type interaction difference for beta rhythm. The same trend is shown with Table 1; beta rhythm has significance on stage and stage-type interaction; alpha has significance on stage; delta and theta have significance on stage-type interaction. Alpha and beta rhythm significant channels are uniformly distributed around the whole brain region, not on a limited brain region. This means that the EEG activity aroused by massage affects the whole brain. EEG alpha power asymmetry results are listed in Table 3. There are no significant differences among stage variations. From the data shown in Table 4, it is obvious that around coherence value is highly related to EEG channel distance. The further each around channel pair becomes, the lower the coherence (Fz-Cz > Fz-Pz > Fz-Oz and Cz-Pz > Cz-Oz) is, and vice versa. To analyze the dependent variable coherence between stages, there was no significant difference for delta rhythm; however, there was a significant difference on the Fz-Cz pair for theta rhythm; there was almost a channel beside Cz-Pz being statistically difference for alpha rhythm; all six channel pairs were statistically different for beta rhythm. The mechanical massage group had more significant differences than the hands-on group for stage coherence variation, as shown in Table 5, which shows the detail average/standard deviation value of around coherence for alpha rhythm.

With further investigation of the coherence change during the four massage durations, D1 to D4, the around coherence testing statistics and the detail around coherence value for the four rhythms are reported in Tables 6 and 7, which are similar to Tables 4 and 5. There were no significant differences for delta and theta rhythms, in both groups. For the alpha rhythm, there was a statistically significant difference on the Cz-Pz pair for the hands-on group. The coherence at D4 session (right Zuo-fei-yu) is higher than the other three sessions. For the alpha rhythm with the mechanical massage group, there was a statistically significant difference on the Fz-Oz pair. The average coherence of D1 session is higher than that of D4 session. The beta rhythm has more coherence variation channels than the other three rhythms. For the hands-on group, there were four channel pairs that were statistically different; they were Fz-Oz, Cz-Pz, Cz-Oz, and Pz-Oz. The coherence at D4 session (right Zuo-fei-yu) is higher than the other three sessions. This result is the same as the alpha rhythm. For the mechanical massage group, there were five channels pairs statistically different; they were Fz-Oz, Fz-Pz, Cz-Pz, Cz-Oz, and Pz-Oz. In summary, the alpha and beta rhythm coherence differences are significant for the four massage sessions in both groups.

Left-right symmetry coherence of the five electrode pairs is illustrated in Tables 8 and 9, which are similar to Tables 4 and 6. For both the hands-on group and the mechanical massage group, the symmetry coherence value of the front brain region is larger than the other channel pairs (hands-on group: FP1-FP2 > F3-F4 > C3-C4 > P3-P4; O1-O2 > P3-P4. mechanical massage group: FP1-FP2 > F3-F4 > C3-C4 > P3-P4; O1-O2 > P3-P4). Higher coherence means higher correlation between channel pairs. For the hands-on group, there was a significant difference for theta rhythm on O1-O2, alpha rhythm on FP1-FP2, F3-F4, and beta rhythm on O1-O2 pairs. For the mechanical massage group, there were more channels with significant difference than the hands-on group. Such as C3-C4 for delta rhythm, F3-F4, C3-C4, and P3-P4 pairs for theta rhythm, F3-F4, C3-C4, P3-P4, and O1-O2 for alpha rhythm, and F3-F4, C3-C4, P3-P4 for beta rhythms. Detail symmetry coherence during the four massage sessions is shown in Table 9. The hands-on group shows significant difference for delta, alpha, and beta rhythmsm, while the mechanical massage group has significant differences in theta and beta rhythms. 

## 4. Discussion

The purpose of this study is to investigate the differences in brain activity resulting from massage by a hands-on approach and by a mechanical approach. There are an abundance of massage tools available, such as massage chairs, massage robes, massage pads, and massage shoes. Traditional massage salons are also very popular. The massage technicians mainly use various hands-on massage skills to attract customers. Although there are abundant studies on massage EEG, the brain activity variation due to different massage methods is the main contribution of this study. Data show that while there were massage stage-type interactions for delta and beta rhythms distributed around the whole brain, there were mainly stage differences for alpha rhythms in the central and after-brain. There were no significant differences in either massage types for the four EEG rhythms. The alpha-asymmetry index was increasing averagely above positive during massage sessions for the hands-on group. It is an interesting result that demonstrates a positive emotional experience during hands-on massage.

EEG coherence analysis reveals more information than EEG power analysis for massage. As described in the introduction, EEG coherence is the spatial correlation between two brain regions. There are several interesting results when comparing the around EEG coherence during massage sessions between the hands-on group and mechanical massage group. The first is that coherence value is higher when channel pair distance is greater. This is valid for both groups. Second, the coherence value of the hands-on group is averagely higher than that of the same channel pairs for the mechanical massage group. The hands-on group's coherence does not change significantly during the massage session, but the mechanical massage group's coherence becomes lower, especially with regard to alpha and beta rhythms. Massage by hand seems to maintain EEG channel coherence, while massage by mechanical may interrupt the original brain interaction between different brain regions. For left-right symmetry coherence, P3-P4 pair is the lowest coherence value pairs for both groups. Similar with around coherence, there were more significant variations on theta, alpha, and beta rhythms for mechanical massage than for hands-on massage, and there was a significant massage stage-type interaction on beta rhythm.

Compared with previous studies [19, 20], there is some interesting and novel information revealed from this study. At first point, EEG coherence data of massage analysis is widely used in this study. Second difference from previous research is the performance of alpha asymmetry index. There is no significant stage difference for both groups, but Jones's data had significant alpha asymmetry variation [25]. As for EEG power result, Diego showed that massage groups treated by professional technicians had significantly decreased alpha and beta waves on the frontal lobe [19]. Our data also had the same decreased alpha power, especially in mechanical group. Both alpha and beta rhythm showed massage stage difference, but mainly on the middle and parietal lobe.

As a manual operation apparatus is used in this study, other massage assistant tools can be investigated with similar methods in further studies, such as massage chairs and massage robes. Other factors that may influence massage performance are massage strength and massage acupoints. Subjects with specific diseases may be subjected to suitable massage methods to alleviate their symptoms. One of the major contributions of this study is to reveal the wide research spectrum of massage via EEG analysis, which is not included for traditional massage application such as for sleep disorder and pain release.

## 5. Conclusion

Different massage media will arouse slightly different neurological responses that may be measured by brain wave activity. EEG power and coherence are two main tools to investigate the brain's activities. Hand-based massage is softer than mechanical-based massage. Many tools have been invented for massage, and there are acupoint factors for different subjects. This paper provides an interesting direction for further massage study and provides an interesting and attractive approach.

## Figures and Tables

**Table 1:** Average EEG subband power distribution of all channels for all subjects. Data is presented as mean (standard deviation).

Type	Stage									
	PRE	D1	D2	D3	D4	P3	P6	Type	Stage	Type *stage
*δ*										
Hand	6.81 (2.39)	6.85 (2.35)	7.46 (3.21)	8.12 (4.11)	8.82 (3.73)	8.66 (3.50)	9.23 (4.31)	n.s.	n.s.	**
Mechanical	9.04 (4.33)	10.16 (6.29)	9.22 (4.87)	8.96 (4.45)	8.36 (3.47)	7.60 (3.42)	7.97 (3.38)			
*θ*										
Hand	4.94 (2.02)	4.60 (1.93)	4.70 (1.82)	4.97 (2.46)	5.23 (2.33)	5.68 (2.22)	5.93 (2.52)	n.s.	n.s.	*
Mechanical	6.56 (3.91)	6.07 (2.97)	6.04 (3.36)	5.81 (3.30)	5.16 (2.15)	5.32 (2.99)	5.45 (2.96)			
*α*										
Hand	10.79 (8.24)	14.57 (9.66)	10.59 (7.71)	8.84 (7.91)	9.30 (9.69)	9.49 (9.71)	9.02 (9.12)	n.s	***	n.s
Mechanical	23.01 (21.55)	23.75 (18.85)	21.63 (18.17)	20.78 (17.48)	19.33 (16.84)	13.47 (13.69)	11.20 (11.69)			
*β*										
Hand	1.28 (0.99)	1.54 (1.21)	1.44 (1.14)	1.54 (1.42)	1.56 (1.31)	1.50 (1.36)	1.55 (1.36)	n.s.	***	**
Mechanical	1.47 (0.80)	1.76 (1.00)	1.76 (1.08)	2.08 (1.45)	2.07 (1.42)	1.27 (0.71)	1.30 (0.69)			

n.s.: Not significant; *P < 0.05; **P < 0.01; ***P < 0.001.

**Table 2:** Stage and type interaction distribution on subband power of each channel. ○ denoted statistical interaction; × denoted no statistical interaction.

Band	Channel														
	FP1	FP2	F7	F3	FZ	F4	F8	FT7	FC3	FCZ	FC4	FT8	T3	C3	CZ
*δ*															
Stage	×	×	×	×	×	×	×	×	×	×	×	×	×	×	×
Type	×	×	×	×	×	×	×	×	×	×	×	×	×	×	×
Stage*type	×	○	×	○	○	○	○	○	○	×	○	○	×	○	×
*θ*															
Stage	×	×	×	×	×	×	×	×	×	×	×	×	×	×	×
Type	×	×	×	×	×	×	×	×	×	×	×	×	×	×	×
Stage*type	×	×	×	×	×	×	○	×	×	×	×	○	×	×	×
*α*															
Stage	×	×	○	×	×	×	×	○	×	×	×	○	○	×	○
Type	×	×	○	×	×	×	×	×	×	○	×	×	×	×	×
Stage*type	×	×	×	×	×	×	×	×	×	×	×	×	○	×	×
*β*															
Stage	○	○	○	×	×	×	○	○	×	×	×	○	○	×	×
Type	×	×	×	×	×	×	×	×	×	×	×	×	×	×	×
Stage*type	○	○	×	×	×	×	○	×	×	×	○	×	×	×	○

Band	Channel														
	C4	T4	TP7	CP3	CPZ	CP4	TP8	T5	P3	PZ	P4	T6	O1	OZ	O2

*δ*															
Stage	×	×	×	×	×	×	×	×	×	×	×	×	×	×	○
Type	×	×	×	×	×	×	×	×	×	×	×	×	×	×	×
Stage*type	○	×	×	×	○	○	×	×	×	○	○	○	×	×	○
*θ*															
Stage	×	×	×	×	×	×	×	×	×	×	×	×	×	×	×
Type	×	×	×	×	×	×	×	×	×	×	×	×	×	×	×
Stage*type	×	×	○	×	○	×	×	×	×	○	×	×	○	×	×
*α*															
Stage	×	○	×	○	○	○	○	×	○	○	○	○	○	○	○
Type	×	×	×	×	×	×	×	○	×	×	×	○	×	×	×
Stage*type	×	×	×	×	×	×	×	×	×	×	×	○	○	○	×
*β*															
Stage	×	×	○	○	○	○	○	○	○	○	○	○	×	○	○
Type	×	×	×	×	×	×	×	×	×	×	×	×	×	×	×
Stage*type	○	×	○	○	○	○	○	○	○	○	○	○	×	×	×

**Table 3:** EEG alpha asymmetry index based on difference power between F3 and F4 channel. ANOVA analysis of seven stages is also estimated. Negative average asymmetry index at each stage is marked in bold.

Method	PRE	D1	D2	D3	D4	P3	P6	*P*-value
Hand								
Mean	**−0.011 **	0.002	0.003	0.019	0.001	**−0.001 **	0.005	0.078
S.D	0.088	0.085	0.081	0.069	0.070	0.071	0.066	
Mechanical								
Mean	0.001	0.005	**−0.009 **	0.003	**−0.006 **	0.010	0.015	0.062
S.D	0.035	0.033	0.039	0.040	0.045	0.038	0.034	

**Table 4:** Stage testing results of around coherence. EEG rhythms and two experiment methods are examined.

Type	Channel					
	FZ-CZ	FZ-PZ	FZ-OZ	CZ-PZ	CZ-OZ	PZ-OZ
*δ*						
Hand	n.s.	n.s.	n.s.	n.s.	n.s.	n.s.
Mechanical	n.s.	n.s.	n.s.	n.s.	n.s.	n.s.
*θ*						
Hand	n.s.	n.s.	n.s.	n.s.	n.s.	n.s.
Mechanical	*	n.s.	n.s.	n.s.	n.s.	n.s.
*α*						
Hand	n.s.	n.s.	n.s.	n.s.	n.s.	n.s.
Mechanical	***	*	***	n.s.	*	**
*β*						
Hand	n.s.	n.s.	n.s.	n.s.	n.s.	n.s.
Mechanical	***	**	*	**	*	**

n.s.: Not significant; *P < 0.05; **P < 0.01; ***P < 0.001.

**Table 5:** Detail average (standard deviation) value of around coherence for alpha rhythm.

Channel	PRE	D1	D2	D3	D4	P3	P6	*P* value
FZ-CZ								
Hand	0.74 (0.13)	0.77 (0.07)	0.77 (0.08)	0.76 (0.07)	0.74 (0.10)	0.71 (0.10)	0.72 (0.10)	0.306
Mechanical	0.72 (0.08)	0.77 (0.07)	0.77 (0.07)	0.79 (0.05)	0.78 (0.06)	0.70 (0.07)	0.70 (0.07)	0.000***
FZ-PZ								
Hand	0.32 (0.22)	0.29 (0.12)	0.30 (0.13)	0.29 (0.09)	0.32 (0.14)	0.29 (0.14)	0.30 (0.18)	0.983
Mechanical	0.18 (0.11)	0.22 (0.12)	0.22 (0.12)	0.24 (0.11)	0.24 (0.11)	0.17 (0.09)	0.21 (0.08)	0.011*
FZ-OZ								
Hand	0.17 (0.25)	0.13 (0.06)	0.12 (0.05)	0.09 (0.07)	0.12 (0.12)	0.11 (0.12)	0.11 (0.15)	0.736
Mechanical	0.15 (0.10)	0.15 (0.09)	0.13 (0.08)	0.13 (0.08)	0.12 (0.06)	0.08 (0.05)	0.06 (0.04)	0.000***
CZ-PZ								
Hand	0.62 (0.15)	0.57 (0.13)	0.57 (0.14)	0.59 (0.09)	0.62 (0.11)	0.61 (0.09)	0.59 (0.11)	0.225
Mechanical	0.48 (0.15)	0.50 (0.15)	0.50 (0.15)	0.52 (0.13)	0.53 (0.13)	0.51 (0.09)	0.55 (0.08)	0.100
CZ-OZ								
Hand	0.23 (0.26)	0.13 (0.06)	0.14 (0.08)	0.13 (0.07)	0.18 (0.12)	0.16 (0.11)	0.14 (0.10)	0.268
Mechanical	0.13 (0.06)	0.14 (0.05)	0.11 (0.04)	0.13 (0.05)	0.13 (0.04)	0.09 (0.03)	0.10 (0.03)	0.039*
PZ-OZ								
Hand	0.52 (0.19)	0.47 (0.13)	0.46 (0.12)	0.46 (0.14)	0.48 (0.14)	0.47 (0.12)	0.47 (0.12)	0.449
Mechanical	0.50 (0.12)	0.51 (0.12)	0.49 (0.09)	0.48 (0.09)	0.47 (0.07)	0.41 (0.16)	0.45 (0.07)	0.009**

**Table 6:** Around coherence testing between acupuncture stages (D1 to D4).

Type			Channel			
	FZ-CZ	FZ-PZ	FZ-OZ	CZ-PZ	CZ-OZ	PZ-OZ
*δ*						
Hand	n.s.	n.s.	n.s.	n.s.	n.s.	n.s.
Mechanical	n.s.	n.s.	n.s.	n.s.	n.s.	n.s.
*θ*						
Hand	n.s.	n.s.	n.s.	n.s.	n.s.	n.s.
Mechanical	n.s.	n.s.	n.s.	n.s.	n.s.	n.s.
*α*						
Hand	n.s.	n.s.	n.s.	**	n.s.	n.s.
Mechanical	n.s.	n.s.	*	n.s.	n.s.	n.s.
*β*						
Hand	n.s.	n.s.	***	*	***	***
Mechanical	*	*	n.s.	*	*	*

n.s.: Not significant; *P < 0.05; **P < 0.01; ***P < 0.001.

**Table 7:** Detail average (standard deviation) value of around coherence testing between acupuncture stages (D1 to D4).

Channel	Left (Jian-wai-yu)	Right (Jian-wai-yu)	Left (Zuo-fei-yu)	Right (Zuo-fei-yu)	*P* value
FZ-CZ					
Hand	0.76 (0.07)	0.74 (0.07)	0.73 (0.08)	0.74 (0.08)	0.066
Mechanical	0.80 (0.04)	0.79 (0.05)	0.82 (0.05)	0.80 (0.06)	0.027*
FZ-PZ					
Hand	0.31 (0.14)	0.33 (0.15)	0.32 (0.14)	0.36 (0.16)	0.065
Mechanical	0.38 (0.13)	0.38 (0.10)	0.44 (0.10)	0.42 (0.09)	0.039*
FZ-OZ					
Hand	0.08 (0.07)	0.09 (0.10)	0.10 (0.08)	0.14 (0.12)	0.001***
Mechanical	0.18 (0.07)	0.18 (0.07)	0.24 (0.07)	0.23 (0.10)	0.073
CZ-PZ					
Hand	0.58 (0.11)	0.59 (0.11)	0.60 (0.10)	0.63 (0.10)	0.033*
Mechanical	0.64 (0.10)	0.64 (0.08)	0.68 (0.08)	0.67 (0.06)	0.043*
CZ-OZ					
Hand	0.15 (0.09)	0.16 (0.12)	0.17 (0.10)	0.23 (0.13)	0.000***
Mechanical	0.27 (0.08)	0.28 (0.09)	0.34 (0.08)	0.32 (0.08)	0.025*
PZ-OZ					
Hand	0.43 (0.12)	0.43 (0.14)	0.46 (0.13)	0.50 (0.12)	0.001***
Mechanical	0.56 (0.05)	0.56 (0.07)	0.61 (0.07)	0.59 (0.08)	0.038*

**Table 8:** Stage testing results of left-right symmetry around coherence.

Type	Channel				
	FP1-FP2	F3-F4	C3-C4	P3-P4	O1-O2
*δ*					
Hand	n.s.	n.s.	n.s.	n.s.	n.s.
Mechanical	n.s.	n.s.	*	n.s.	n.s.
*θ*					
Hand	n.s.	n.s.	n.s.	n.s.	*
Mechanical	n.s.	*	***	**	*
*α*					
Hand	*	*	n.s.	n.s.	n.s.
Mechanical	n.s.	***	***	**	***
*β*					
Hand	n.s.	n.s.	n.s.	n.s.	*
Mechanical	n.s.	***	***	*	n.s.

**Table 9:** Left-right symmetry coherence testing between acupuncture stages (D1 to D4).

Type	Channel				
	FP1-FP2	F3-F4	C3-C4	P3-P4	O1-O2
*δ*					
Hand	n.s.	n.s.	n.s.	**	n.s.
Mechanical	n.s.	n.s.	n.s.	n.s.	n.s.
*θ*					
Hand	n.s.	n.s.	n.s.	n.s.	n.s.
Mechanical	n.s.	*	n.s.	*	n.s.
*α*					
Hand	*	*	n.s.	n.s.	n.s.
Mechanical	n.s.	n.s.	n.s.	n.s.	n.s.
*β*					
Hand	n.s.	n.s.	n.s.	***	n.s.
Mechanical	n.s.	*	**	*	n.s.

## References

[1] Bonevski B, Wilson A, Henry DA (2008). An analysis of news media coverage of complementary and alternative medicine. *PLoS ONE*.

[2] Beider S, Moyer CA (2007). Randomized controlled trials of pediatric massage: a review. *Evidence-Based Complementary and Alternative Medicine*.

[3] Field TM (1998). Massage therapy effects. *American Psychologist*.

[4] Maxwell-Hudson C, Lousada S (2001). *Massage: The Definitive Visual Reference*.

[5] Frances MT (1993). *Healing Massage Techniques: Holistic, Classic, and Emerging Methods*.

[6] Mehling WE, Gopisetty V, Daubenmier J, Price CJ, Hecht FM, Stewart A (2009). Body awareness: construct and self-report measures. *PLoS ONE*.

[7] Ellis V, Hill J, Campbell H (1995). Strengthening the family unit through the healing power of massage. *American Journal of Hospice & Palliative Care*.

[8] Kresse MC (1996). Getting a leg up on sequential compression therapy. *Nursing*.

[9] Moraska A, Pollini RA, Boulanger K, Brooks MZ, Teitlebaum L (2010). Physiological adjustments to stress measures following massage therapy: a review of the literature. *Evidence-Based Complementary and Alternative Medicine*.

[10] Tsao JCI (2007). Effectiveness of massage therapy for chronic, non-malignant pain: a review. *Evidence-Based Complementary and Alternative Medicine*.

[11] Dibble SL, Chapman J, Mack KA, Shih AS (2000). Acupressure for nausea: results of a pilot study. *Oncology Nursing Forum*.

[12] Livingston K, Beider S, Kant AJ, Gallardo CC, Joseph MH, Gold JI (2009). Touch and massage for medically fragile infants. *Evidence-Based Complementary and Alternative Medicine*.

[13] Hur MH, Yang YS, Lee MS (2008). Aromatherapy massage affects menopausal symptoms in Korean climacteric women: a pilot-controlled clinical trial. *Evidence-Based Complementary and Alternative Medicine*.

[14] Kuriyama H, Watanabe S, Nakaya T (2005). Immunological and psychological benefits of aromatherapy massage. *Evidence-Based Complementary and Alternative Medicine*.

[15] Hatayama T, Kitamura S, Tamura C, Nagano M, Ohnuki K (2008). The facial massage reduced anxiety and negative mood status, and increased sympathetic nervous activity. *Biomedical Research*.

[16] Ejindu A (2007). The effects of foot and facial massage on sleep induction, blood pressure, pulse and respiratory rate: crossover pilot study. *Complementary Therapies in Clinical Practice*.

[17] Webster JG (1997). *Medical Instrumentation: Application and Design*.

[18] Jones NA, Field T (1999). Massage and music therapies attenuate frontal EEG asymmetry in depressed adolescents. *Adolescence*.

[19] Diego MA, Field T, Sanders C, Hernandez-Reif M (2004). Massage therapy of moderate and light pressure and vibrator effects on EEG and heart rate. *International Journal of Neuroscience*.

[20] Field T, Ironson G, Scafidi F (1996). Massage therapy reduces anxiety and enhances EEG pattern of alertness and math computations. *International Journal of Neuroscience*.

[21] Field T, Diego MA, Hernandez-Reif M, Deeds O, Figuereido B (2006). Moderate versus light pressure massage therapy leads to greater weight gain in preterm infants. *Infant Behavior and Development*.

[22] Davidson RJ (1998). Anterior electrophysiological asymmetries, emotion, and depression: conceptual and methodological conundrums. *Psychophysiology*.

[23] Davidson RJ (2000). Affective style, psychopathology, and resilience: brain mechanisms and plasticity. *American Psychologist*.

[24] Klimesch W (1997). EEG-alpha rhythms and memory processes. *International Journal of Psychophysiology*.

[25] Jones NA (1998). Massage therapy attenuates right frontal EEG asymmetry in one-month-old infants of depressed mothers. *Infant Behavior and Development*.

[26] Nunez PL, Silberstein RB, Shi Z (1999). EEG coherency II: experimental comparisons of multiple measures. *Clinical Neurophysiology*.

[27] Nunez PL, Srinivasan R, Westdorp AF (1997). EEG coherency I: statistics, reference electrode, volume conduction, Laplacians, cortical imaging, and interpretation at multiple scales. *Electroencephalography and Clinical Neurophysiology*.

[28] Ahn AC, Colbert AP, Anderson BJ (2008). Electrical properties of acupuncture points and meridians: a systematic review. *Bioelectromagnetics*.

[29] Colbert AP, Yun J, Larsen A, Edinger T, Gregory WL, Thong T (2008). Skin impedance measurements for acupuncture research: development of a continuous recording system. *Evidence-Based Complementary and Alternative Medicine*.

[30] McKechnie A, Wilson F, Watson NL, Scott D (1983). Anxiety states: a preliminary report on the value of connective tissue massage. *Journal of Psychosomatic Research*.

[31] Jodo E, Yamada Y, Hatayama T (1988). Effects of facial massage on the spontaneous EEG. *Tohoku Psychologica Folia*.

